# THERAPEUTIC EXPECTATIONS AND INTEREST IN END-OF-LIFE CARE: ROLE OF AGEISM, AGING ANXIETY, AND DEATH ANXIETY

**DOI:** 10.1093/geroni/igae098.2393

**Published:** 2024-12-31

**Authors:** Eve Root, Grace Caskie

**Affiliations:** Lehigh University, Clayton, Missouri, United States; Lehigh University, Bethlehem, Pennsylvania, United States

## Abstract

The field of mental health is not yet equipped to meet the needs of the growing older adult population. More psychologists are needed in the field of geropsychology across all specialties, including end-of-life care. However, no known research has examined psychology trainees’ attitudes or interest in providing end-of-life care to older adults. Under the frame of Terror Management Theory (Martens et al., 2005), this study examined the relationship of ageism, aging anxiety, and death anxiety with psychology trainees’ therapeutic expectations and interest in engaging in end-of-life work with older adults. Using structural equation modeling, a model was tested to examine ageism, aging anxiety, and death anxiety as independent predictors of interest and therapeutic expectations towards end-of-life care with individuals in later life. In a sample of 197 psychology doctoral students enrolled in an APA-accredited program, the model showed good fit after modifications (CFI =.92, TLI =.90, RMSEA =.08, SRMR =.09). Higher aging anxiety predicted lower interest working with this population as well as worse therapeutic expectations. Ageism was not significantly related to therapeutic expectations but was positively related to interest. Death anxiety did not significantly predict either outcome variable. These findings suggest training programs and early clinical opportunities should consider the role of existential anxiety, particularly aging anxiety, as well as ageism’s impact on trainees’ career interests and perceptions towards working with older adults living with life-limiting illness.

